# Case report: increased single-nephron estimated glomerular filtration rate in an adult patient with low birth weight

**DOI:** 10.1186/s12882-020-01728-6

**Published:** 2020-03-04

**Authors:** Yuriko Shiozaki, Tomoyuki Fujikura, Shinsuke Isobe, Ibuki Takatsuka, Taichi Sato, Daiki Goto, Sayaka Ishigaki, Naro Ohashi, Hideo Yasuda

**Affiliations:** Hamamatsu University School of Medicine, Internal Medicine 1, 1-20-1 Handayama, East Ward, Hamamatsu, Shizuoka, 431-3192 Japan

**Keywords:** Low birth weight, Single-nephron estimated glomerular filtration rate, Glomerular hyperfiltration

## Abstract

**Background:**

Low birth weight (LBW) is associated with end-stage kidney disease and hypertension and is considered to be a surrogate marker of low nephron number. Low nephron number is hypothesized to contribute to glomerular hyperfiltration that may cause kidney injury; however, this is not yet proven. Until now, the hyperfiltration in LBW patients has not been shown directly yet.

**Case presentation:**

A 23-years-old female was referred with the persistent proteinuria and decreased renal function (estimated glomerular filtration rate by cystatin C (eGFR_cys_); 41.86 ml/min). She was a premature baby with low birth weight (704 g, 24 gestational weeks). Renal biopsy demonstrated focal segmental glomerulosclerosis (FSGS) of the perihilar variant with expanded glomerular diameter. We calculated the single-nephron estimated glomerular filtration rate (SN-eGFR) that was higher than that of the same age group in the healthy living kidney donors and speculated that glomerular hyperfiltration is a pathophysiological cause of FSGS.

**Conclusion:**

This is the first case of SN-eGFR measurement in a patient with LBW. The increased SN-eGFR in this case provides an important insight into the pathophysiological mechanisms of LBW for its progression to kidney disease.

## Background

Low birth weight (LBW) is associated with end-stage kidney disease and hypertension [1, 2], and it is a marker of poor fetal growth because of pre-term birth and/or intrauterine growth restriction. LBW is considered to correlate with reduced nephron number [3]. This reduced nephron number is hypothesized to contribute to the glomerular hyperfiltration, followed by kidney injury [4]. Focal segmental glomerulosclerosis (FSGS) patients who had a history of LBW were shown to have glomerulomegaly, high glomerular volumes, and low glomerular densities [5, 6], which supports the hyperfiltration hypothesis. However, until now, the hyperfiltration in LBW patients has not been shown directly yet.

Single-nephron glomerular filtration rate (SN-GFR) measurement has been proposed using healthy living kidney donors [7], which may provide us important insights into some glomerular diseases. Indeed, the measurement of SN-GFR is anticipated to elucidate the pathophysiological association between glomerular hyperfiltration and LBW [8].

We report a case of FSGS with a history of LBW in which the SN-eGFR is increased, suggestive of glomerular hyperfiltration. Our finding shows the need for a large number of longitudinal SN-GFR measurements in LBW patients to further understand the mechanism of FSGS.

## Case presentation

A 23-years-old female was referred to our hospital following a complaint of persistent proteinuria. Proteinuria (1+) had been detected on routine examinations at the age of 21, and it has been getting worse in the last 2 years. (1 + →2+, 1.54 g of urinary protein in 24 h). She was a premature baby with low birth weight (704 g, 24 gestational weeks). Her growth and development were normal. She has a family history of Werner syndrome on her mother’s side. She had no medication and no history of pregnancy. Physical examination revealed the following: height, 150 cm; weight, 41 kg; body mass index, 17.9 kg/m^2^; blood pressure, 120/58 mmHg; pulse rate, 65 beats/min; and temperature, 36 °C. She had no history of hypertension, her estimated daily salt intake was 5.0–6.0 g. She is not a vegetarian, and her estimated daily protein intake was 45.0 g. There was no abnormality observed in other physical examinations.

Urinalysis revealed proteinuria (2+) but no hematuria. Her 24-h urinary protein, serum creatinine, and serum cystatin C levels were 1.08 g, 0.94 mg/dL, and 1.39 mg/L, respectively. Her serum albumin was 4.4 g/dl. Her eGFR with body surface area (BSA) adjustment was calculated: eGFR_cys_ calculated using CKD-EPI (The Chronic Kidney Disease Epidemiology Collaboration) cystatin C equation, 55 ml/min/1.73 m^2^ [9]; eGFR_cr-cys_ calculated using the modified CKD-EPI creatinine- cystatin C equation with the coefficient for Japanese (0.908 × CKD-EPI_cr-cys_) [10], 60 ml/min/1.73 m^2^; and eGFR_cr_ calculated using the equation for the Japanese population, 62 ml/min/1.73 m^2^ [11]. Without BSA adjustment, individualized eGFR_cys_, eGFR_cr-cys_, and eGFR_cr_ were 41.86 ml/min, 45.61 ml/min, and 47.19 ml/min, respectively (Table 1). Creatinine clearance was 47.87 ml/min. On ultrasound, the longitudinal diameters of her kidneys were 9.9 cm for the right kidney and 9.2 cm for the left kidney.

**Table 1:** Detailed morphometric characteristics and comparison with the other two studies

Characteristics	This study	Living kidney donors (93–94% Caucasian)	Living kidney donors (100% Japanese)^***^
Kidney function			
eGFR_cys_ (ml/min)	41.86	Total GFR: 127 ± 25 ml/min^*^	eGFR_cr_: 76 ± 12 mL/min/1.73 m^2^
eGFR_cr-cys_ (ml/min)	45.61		
eGFR_cr_ (ml/min)	47.19		
Morphometric measurements			
Renal parenchymal volume per kidney (cm^3^)	90.15		124 ± 24
Renal cortical volume per kidney (cm^3^)	69.25		89 ± 19
Cortical area in biopsy specimen (mm^2^)	8.62		2.96 ± 0.79
Glomerular volume (× 10^6^ μm^3^)	7.16		2.44 ± 1.04
Glomerular density (/mm^3^)	1.33		2.42 ± 0.67
Non-sclerotic nephron number per kidney	208,000		650,000 ± 220,000
% Globally sclerotic glomeruli	20%	1.1 (0.8–1.5)^**^	4.7 (0–35.3)
Total nephron number per kidney	265,000	970,000 ± 430,000^*^	710,000 ± 220,000
Calculated nephron-related values			
Single-nephron eGFR_cys_ (nl/min)	101	Single-Nephron GFR: 79 ± 42 nl/min^*^	
Single-nephron eGFR_cr-cys_ (nl/min)	110		
Single-nephron eGFR_cr_ (nl/min)	113		

eGFR_cys_ with body surface area (BSA) adjustment calculated by the cystatin C level using CKD-EPI_cys_, and eGFR_cr-cys_ with BSA adjustment calculated by both the creatinine and cystatin C levels using CKD-EPI_cr-cys_ with the coefficient modification using the Japanese eq. (0.908 ×) [11]. eGFR_cr_ with BSA adjustment calculated by the creatinine level using the equation for the Japanese population [10]. eGFR_cys_, eGFR_cr-cys_, and eGFR_cr_ are individualized without BSA adjustment. Single-nephron eGFR_cys_, eGFR_cr-cys_, and eGFR_cr_ are calculated from the individualized eGFR_cys_, eGFR_cr-cys_, and eGFR_cr_, respectively

^*^The data of the same age group (18–29 years) in the healthy living kidney donors (93.7% Caucasian) [7]

^**^The data of the same age group (18–29 years) in the healthy living kidney donors (92.7% Caucasian) [12]

^***^The data in the healthy living kidney donors (100% Japanese, 56.7 ± 9.5 years) [13]

**Fig. 1:**
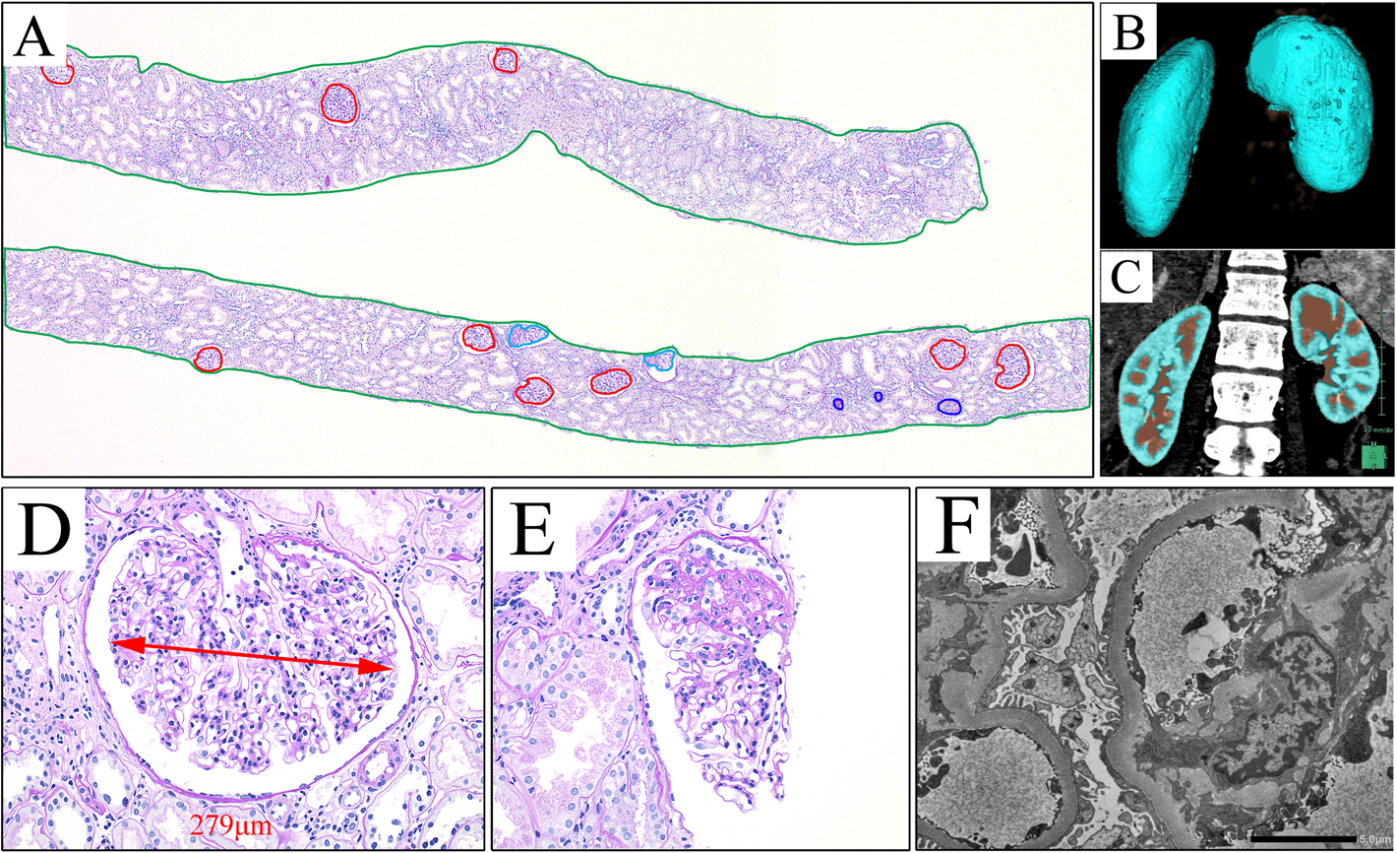
Kidney biopsy morphometry and computed tomography (CT) scan with contrast. **a** The non-sclerotic glomerular (red outline), partial non-sclerotic glomerular (cyan outline, counted as 0.5 glomerulus), and total glomerular areas (both red, cyan, and blue outlines) were measured. The glomerular volume and density were measured from the area of the glomeruli and the area of the cortex (green outline). **b** A three-dimensional image was reconstructed to determine the cortical volume. **c** The enhanced area in the coronal plane of the CT scan was semiautomatically colored. **d**, **e** Representative glomeruli are shown. Segmental sclerosis at perihilar location is noted (**e**). **f** Electron microscopy reveals minimal foot process effacement

A percutaneous renal biopsy was performed to establish the diagnosis. On microscopy, the specimen contained 15 glomeruli. Three glomeruli were globally sclerotic, which showed a solidification pattern. Two glomeruli had segmental sclerosis of the perihilar variant with a hyalinosis. The mean of glomerular diameter of 15 glomeruli was 233 μm (Fig. 1). Electron microscopy revealed minimal foot process effacement and no electron-dense deposits (Fig. 1).

We calculated the single-nephron estimated glomerular filtration rate (SN-eGFR) using the following method. Using contrast computed tomography (CT) angiograms, the patient’s kidneys were three-dimensionally reconstructed, and total renal cortical volume was estimated using SYNAPSE VINCENT (FUJIFILM, Japan). Glomerular area (mm^2^), glomerular volume (× 10^6^ μm^3^), and glomerular density (/mm^3^) were measured using the method reported by Denic et al. [12] (Fig. 1 a). Considering that the biopsy specimen had two segmentally sclerotic glomeruli, we included them into the non-sclerotic glomerular number. The total glomerular number and non-sclerotic glomerular number per kidney were calculated by multiplying the total cortical volume (mm^3^) by the glomerular density (/mm^3^), and dividing by 2 (per kidney), dividing by 1.43 (tissue volume shrinkage due to paraffin embedding), dividing by 1.268 (volume shrinkage due to loss of tissue perfusion pressure), and rounded to the nearest 10,000 nephrons [12]. The percentage of global glomerulosclerosis was calculated by dividing the globally sclerotic glomeruli by the total glomerular number (Table 1). The SN-eGFR was calculated as the individualized eGFR divided by the total nephron number for both kidneys and rounded to the nearest 1 nl/min [7]. We found out that her SN-eGFR was higher than that of the same age group (79 ± 42 nl/min) [7] (Table 1).

After the diagnosis, angiotensin receptor blocker (losartan 50 mg) was administered. At follow-up 6 months later, the 24-h urinary protein decreased slightly (0.87 g) without alterations in serum creatinine (0.91 mg/dl).

## Discussion and conclusions

We report an adult case of FSGS with LBW and demonstrate a higher SN-eGFR than that of the age-matched healthy living kidney donors [7] (Table 1). Increased SN-eGFR could indicate glomerular hyperfiltration, which contributes to the pathophysiology of FSGS.

Glomerular hyperfiltration caused by low nephron number is hypothesized to be the pathophysiology of kidney diseases. In fact, some epidemiologic studies have shown the association between LBW and comorbidities of kidney diseases in childhood [14] or adolescence [15]. In Helsinki Birth Cohort Study, LBW and prematurity were shown to be associated with increased risk for the development of chronic kidney disease in adulthood [16]. Using the two-dimensional areal density of the glomeruli calculated using the kidney biopsy specimens, LBW-FSGS children had lower glomerular density and larger glomerular volume than the normal-birth-weight FSGS children and exhibited the histopathological and clinical findings that were consistent with secondary FSGS (e.g. perihilar variant, mild proteinuria) [5]. These studies support the pathophysiology of glomerular hyperfiltration that is associated with LBW.

Recently, using both CT angiography and kidney biopsy specimen, the novel methods for estimating the total nephron number in living humans have been proposed [12, 13]. Furthermore, combining this estimation of the total nephron number and measurement of GFR, Denic et al. showed the measurement of SN-GFR in healthy living kidney donors [7]. Using this same technique with eGFR, we estimated the SN-eGFR in this case, which was higher than that of the same age group in the healthy living kidney donors (93.7% Caucasian) [7] (Table 1). Some biases on the measurement of total nephron number or eGFR can prevent us from the accurate interpretation of SN-eGFR. From several human population studies, the average nephron number varies up to 13-fold, and the mean glomerular volume varies up to 7-fold [17]; hence, the genetic difference between the healthy living kidney donors (93.7% Caucasian) [7] and a Japanese patient could influence the nephron number. However, the estimated total nephron number in this case is much lower than the Japanese living kidney donors (265,000 vs. 710,000 ± 220,000 per kidney) [13] (Table 1). Since we did not use an accurate GFR measurement such as inulin clearance, the inaccuracy of estimating eGFR may mislead the measurement of SN-eGFR. Yet, the two equations used to estimate eGFR (eGFR_cys_ and eGFR_cr-cys_) here are based on CKD-EPI study [9] and are developed to establish a more diagnostic accuracy in the Japanese population [11], and both results are significantly associated with each other. Accordingly, we consider that the estimated SN-eGFR in this case is reliable and considerably high.

Occasionally, glomerular hyperfiltration is a compensatory mechanism, such as in age-related nephrosclerosis [12] or hypertension-related nephrosclerosis, of an acquired nephron loss. The ratio of globally sclerotic glomeruli in this case (20%) is higher than those of healthy living kidney donors (Table 1) [12, 13], and they showed the “solidification” pattern of global sclerosis, and the patient in this case has no history of hypertension. This means her glomerular hyperfiltration is not a result of acquired nephron loss but a result of congenitally low nephron number, which might be associated with LBW. It is certainly unclear whether this glomerular hyperfiltration is indeed pathophysiological cause of FSGS. In general, kidney donors lost about half of the nephrons after the donation, followed by glomerular enlargement with increased SN-GFR as a compensatory mechanism. Nevertheless, kidney donation itself rarely leads to progressive kidney disease. However, in this case, the biopsy findings revealed some segmental sclerotic glomeruli of the perihilar variant and glomerulomegaly, which is consistent with the predominant findings observed in LBW-FSGS patients [6] and obesity-related FSGS patients [18]. Therefore, we consider that the glomerular hyperfiltration experienced in this case could be the pathophysiological cause of FSGS, although its detailed pathophysiological mechanism is not yet proven.

Although this case study can directly show neither the association between glomerular hyperfiltration and LBW nor the pathophysiology of glomerular hyperfiltration, this study could provide an important insight into the pathophysiological mechanisms of LBW for its progression to kidney disease. To specifically elucidate the pathophysiology of glomerular hyperfiltration, large and longitudinal studies are required. Additionally, instead of performing a kidney biopsy that is an invasive procedure, the development of non-invasive procedures to estimate nephron number [19] may hasten the elucidation of this mechanism.

## Data Availability

All the data relevant to this report are included in the manuscript.

## Abbreviations

CKD-EPI: The Chronic Kidney Disease Epidemiology Collaboration
FSGS: Focal segmental glomerulosclerosis
LBW: Low birth weight
SN-eGFR: Single-nephron estimated glomerular filtration rate
SN-GFR: Single-nephron glomerular filtration rate
